# Factors Associated with Intention to Use Digital Mental Health Interventions Among AANHPI Emerging Adults in the United States: Application of the Seeking Mental Health Care Model

**DOI:** 10.21203/rs.3.rs-9285173/v1

**Published:** 2026-04-24

**Authors:** John Bunyi, Shinyi Wu, Angel Hsing-Chi Hwang, Hans Oh

**Affiliations:** University of Southern California

**Keywords:** AANHPI, Digital mental health interventions, Help-seeking, Mental health literacy, Perceived stigma, Emerging adults, Seeking Mental Health Care model, Health disparities, Intention to use technology, Multivariable logistic regression

## Abstract

Asian American, Native Hawaiian, and Pacific Islander emerging adults significantly underutilize traditional face-to-face mental health services despite experiencing prevalence of mental illness comparable to the general population. Digital mental health interventions offer a promising avenue for improving access for this "digitally native" population. This study utilizes the Seeking Mental Health Care model to examine the individual-level psychological and demographic factors associated with the intention to use digital mental health interventions among a diverse sample of Asian American, Native Hawaiian, and Pacific Islander emerging adults. Using multivariable logistic regression, key predictors included perceived need for support and mental health literacy, while controlling for symptom severity (anxiety and depression) and sociodemographic variables. A higher perceived public stigma was found to be associated with a greater likelihood of intending to use digital mental health interventions. Findings reveal that individual factors, such as higher perceived need and mental health literacy, are significantly associated with a greater intention to use digital mental health interventions, supporting the utility of using the Seeking Mental Health Care model to better understand the adoption of digital interventions in this underserved population, while suggesting other points of intervention such as psychoeducation for greater mental health literacy that could lead to greater treatment engagement.

## BACKGROUND AND INTRODUCTION

### Growing mental health needs among Emerging Adults

Emerging Adults in the U.S., defined as those between the ages of 18–29 years old, are increasingly in need of support for their mental health. Recent studies have suggested that amidst a general rise in mental illness, anxiety and depression have increased in prevalence among emerging adults (1, 2, 3). These mental health challenges may stem from a range of factors, including key transitional events across the lifespan such as frequent changes in personal relationships, repeated involuntary job shifts, relocation to unfamiliar environments, and financial instability (4, 5). Additionally, biological influences like ongoing brain development and sociocultural influences like increasing connectedness to the internet, may play a role in how one comes to understand these transitional events (6). Adding to the rise in need, mental health help-seeking in this group is lacking and access to care for this group is limited (7).

### AANHPI access to mental health care

AANHPI emerging adults face social factors that can affect their approach to mental health. Contrary to the “model minority” stereotype (8) which portrays AANHPI individuals as high achievers with minimal mental health needs, AANHPI individuals experience rates of mental illness that are comparable to or in some cases worse than, the general population (9, 10, 11, 12). Over 30% of AANHPI individuals ages 18–25 experienced some form of mental illness in 2024 (12). Among AANHPI individuals, rates of serious outcomes of mental illness, such as substance use and suicide, are rising (13, 14). Some literature suggests that these rates may also be reflective of underreporting due to the aforementioned model minority stereotype or possibly due to ethno-racial differences in subjective evaluations of impairment, resulting in lower rates of clinical diagnoses (15). More concerning, however, is that despite exhibiting comparable rates of mental illness, AANHPI are much less likely to seek formal support for their mental health (14, 16).

### Digital access and Digital mental health interventions (DMHI) among AANHPI

DMHIs pose a promising solution to mental health care barriers faced by AANHPI emerging adults. For one, AANHPI emerging adults are more likely to have grown up as “digital natives” and be much more likely to turn to digital tools for support (17). Asian Americans are more likely to purchase a new smartphone than the general population (18), and AANHPI individuals access smartphones at high rates for reasons outside of mental health needs such as communicating with family and friends, intergenerational connection and better care of elders and dependent family members, and media consumption (18).

Given how digitally connected AANHPI emerging adults are, DMHIs present an opportunity to meet AANHPI where they are. Broadly defined, DMHIs span a variety of technologies. Smartphone applications, or “apps” are a relatively mature platform for connecting users to support (19) and for delivering mental health interventions (20, 21), such as cognitive behavioral therapy (CBT), mindfulness, and psychoeducation (20, 21). Recent literature suggests that certain products can be effective and scalable (19, 22, 23, 24). Users across studies have often cited anonymity, convenience and availability, and potential cost savings as reasons for using apps (25).

DMHIs may also include technology platforms that are widely available and utilized by many, including but not limited to: SMS/MMS (text and multimedia) messaging, social networking sites, telehealth platforms, websites, and video games geared towards addressing mental health (26, 27). The wide adoption of these technologies by the general population make them promising avenues of delivering mental healthcare. Other platforms to deliver DMHIs include more cutting-edge technologies such as artificial intelligence (AI), virtual reality (VR), and wearable technology (e.g., smartwatches, smart rings, etc.) However, more work is needed to better understand public adoption and perception of these technologies and how people use such tools for their mental health, especially among the AANHPI emerging adult population.

Despite the promise of technology, there is limited understanding of how AANHPI emerging adults view digital mental health. Most literature that study DMHIs in the U.S. have largely studied users who are more representative of those who use commercially available technologies (i.e., White, middle-age women; 28), while research into the use of DMHIs to address AANHPI mental health needs remains limited, despite the development of culturally-tailored DMHIs designed specifically for AANHPI audiences (29, 30). This has resulted in a limited understanding of how such tools perform in the real-world amongst AANHPI and other historically hard-to-reach populations in mental health care. Furthermore, mental health apps have historically seen limited rates of adoption (31, 32, 33), with a large number of users concentrated in the top three to five commercially available apps (34), calling into question the applicability of such interventions for equitably addressing public health needs around mental health. And while users report DMHIs addressing some barriers to access, other factors such as privacy concerns, cost, and the severity of mental health symptoms (25) may hamper adoption.

Altogether, the breadth of technologies available for supporting mental health suggests a wide range of possibilities to improve access to mental health care for AANHPI individuals. For AANHPI emerging adults, who are more likely to be “digital natives” yet face cultural and structural barriers to accessing care, it is critical to understand what mental health help-seeking factors are associated with the intention to use digital tools by real-world users and non-users. Without a clear understanding, equitably implementing DMHI becomes difficult, risking the perpetuation of existing barriers to care or, potentially, worsening disparities in access.

### The Seeking Mental Health Care Model to understanding digital help-seeking

DMHI research has largely been guided by technology acceptance frameworks (25), although there have been attempts to utilize other models to explain the use of health information technologies (35, 36). Given the subjective, episodic, systemic, and social nature of mental illness, it is beneficial to explore what leads people to use DMHIs through the lens of mental health help-seeking theory. This work will utilize the Seeking Mental Health Care Model (SMHC; 37) to explore personal and attitudinal factors that directly affect digital help-seeking and the intention to use DMHIs. These include a self-identified need for mental health care (or *perceived need for support*), *mental health literacy*, and *stigma* towards mental illness. The help-seeking pathways outlined by the SMHC model make it a useful lens for examining what leads to the intention to use DMHI within a real-world sample of AANHPI emerging adults.

### Aims and Hypothesis

The primary aim of this paper is to explore what individual-level factors are associated with the intention to use DMHIs amongst AANHPI in the real-world as guided by the SMHC, a mental health help-seeking framework. This paper will also explore ethnic heterogeneity by examining differences in the intention to use DMHIs across diverse AANHPI ethnic subgroups, controlling for individual psychological (i.e., mental health symptoms) and demographic variables. Based on the SMHC model, the primary guiding hypothesis for this manuscript is that multivariable logistic regression will reveal significant associations between the outcome (intention to use DMHIs) and the independent variables of interest, specifically perceived need for support, mental health literacy, and perceived stigma.

## METHODS

### Study Design and Participants

#### Sampling and Recruitment.

This study utilized survey data from the Personalized Normative Feedback (PNF) Project (PI: Dr. Hans Oh; University of Southern California IRB: APP-24-05114), a survey of Black and AANHPI emerging adults across the United States.

This study focused on the project’s sample of 1,577 AANHPI participants, ages 18–29, living in the United States. Researchers collected data via Qualtrics survey panels from July 2024 to September 2024. Qualtrics survey panels consist of pre-enrolled participants who consent to be contacted for research studies (Qualtrics, Provo, UT). Academic researchers, industry partners, and organizations use these panels to obtain data from target populations. Qualtrics recruits participants through multiple channels, including partner websites, referral systems, curated email lists, and social media outreach.

To ensure sample quality and mitigate risks associated with online recruitment, multiple verification procedures were implemented. Participants were first screened using CAPTCHA technology prior to survey initiation. Qualtrics panel providers, or vendors, also applied their own identity confirmation protocols, which included TrueSample, Verity, SmartSample, USPS address verification, and digital fingerprinting (38). Vendors further cross-verified respondent demographic information, residential addresses, and email accounts to further ensure data integrity. For this study, a total of three vendors were leveraged for recruitment of participants. Participants were incentivized with rewards appropriate to their recruitment pathway (e.g., retail gift cards, customer loyalty points). Informed consent was obtained electronically from all participants at the beginning of the survey.

We counted only participants who completed the full survey toward recruitment quotas (1,200 Black and 1,600 AANHPI respondents). In total, 15,876 individuals initiated the survey; 2,801 completed all items, resulting in a 17.6% completion rate. This observed completion rate falls between previous Qualtrics experiences reported by Miller et al. (39), who distributed two surveys with 13.2% and 26.3% completion rates.

#### Participants.

Despite being one of the largest racial-ethnic groups in the U.S., certain AANHPI ethnic subgroups continue to be hard-to-reach in research recruitment (40, 41, 42) resulting in their continued underrepresentation in mental health research. This may be due to stigma, general distrust of larger systems, or language barriers (40). To ensure adequate representation from at least the largest AANHPI ethnic groups, we set quotas with Qualtrics to sample 200 individuals each from seven ethnic groups (Chinese, Filipino, Indian, Japanese, Korean, NHPI, and Vietnamese) with an eighth category of “Other AANHPI” to capture those who identify with other ethnic groups not listed. Individuals who identified as being a member of multiple AANHPI ethnic groups were recategorized during the analysis as being “Multi-Ethnic,” while those who identified as both AANHPI and another race group (i.e., White, Black, Latinx/e, Middle Eastern, Native American) were recategorized as “Multi-Racial AANHPI.”). This was done to better account for the unique mental health experiences of individuals with multiple ethnic/racial backgrounds (43). The research team's subsequent cleaning and recategorization resulted in a final analytic sample of 1,577 self-identified AANHPI individuals. Although slightly below the initial quota, this process yielded an ethnically diverse AANHPI sample uniquely representative of at least the largest AANHPI ethnic groups.

### Measures and variables

The following variables were utilized to explore individual-level factors associated with the intention to use DMHIs among AANHPI, based on principles of the SMHC model.

The primary outcome variable examined is *intention to use DMHIs*, measured dichotomously using a single self-reported item indicating whether the respondent intends to use a digital mental health tool (defined as “Wellness apps or websites,” ‘Apps or websites for treating depression, anxiety, or other mental health conditions,” “Online or telephone-based therapy services,” “Online support groups,” and/or a self-identified tool).

The main independent variables were derived from the theoretical constructs of the SMHC model. Perceived need for support, was assessed using a single self-reported item taken from the California Health Interview Survey (44) indicating a need for emotional support in the past 12 months. Mental health literacy (MHL) was measured using a reduced 7-item scale adapted from the 26-item Mental Health Literacy Measure (45), assessing knowledge and resource-oriented literacy. Items were dichotomized and combined into a composite score, where higher scores indicated higher mental health literacy ( = 0.76). Measures of perceived *public stigma* (e.g., “Most people would accept a person with mental illness as a close friend,” “Most employers will not hire a person with mental illness.”) and perceived *self-stigma* (e.g., “I would accept a person with mental illness as a close friend,” “I will not hire a person with mental illness.”) were derived from the 24-item Perceived Devaluation and Discrimination Scale (PDDS; 46). These were treated as continuous mean scores, and notably, the scores were reverse-coded such that higher scores indicated lower perceived stigma ( = 0.74 for the public stigma scale and = 0.83 for the self-stigma scale).

In addition to the primary independent variables, the analytical model incorporates several covariates to account for covariates related to symptom experience, treatment engagement, and demographics. The SMHC model frames symptom severity of mental illness and treatment experiences as key antecedents that influence the intention to seek help. Thus, *symptom severity* was controlled for by including continuous scores for both anxiety (GAD-7) and depression (PHQ-9) simultaneously within the model. *Current therapy use*, a proxy for treatment experience, was included as a dichotomous variable (Yes/No). Sociodemographic factors included *age* (continuous), *sex* (dichotomous; Male/Female), *education level* (dichotomous; “At least a bachelors/associate degree”/“No college degree”), *employment status* (dichotomous; Working/Not Working), and *health insurance status* (dichotomous; Insured/Not insured). We also included language spoken at home growing up (dichotomous; “Mostly English to Only English”/“Only non-English to Equally English and non-English”) as a proxy for *acculturation*. Finally, *ethnicity* was included using dummy-coded categories, with the Chinese group serving as the reference category. A dummy-coded variable for *vendor* source was also utilized to control for potential sampling differences across the recruitment panel sources.

### Data Analysis

Analyses began with descriptive statistics to characterize the study sample in terms of sociodemographic characteristics, mental health symptoms, and individual help-seeking variables. A preliminary power analysis confirmed sufficient statistical power for the logistic regression model. Consistent with the data collection methodology, the final dataset contained no missing values.

Multivariable logistic regression was employed to estimate the associations between individual-level factors and the intention to use DMHI. The final model included the main SMHC variables and key covariates. Additional sensitivity analyses were performed to ensure robustness of the findings. To do so, we reran the primary model while excluding participants recruited through Vendor 3 (n = 374) after preliminary analyses indicated Vendor 3 participants exhibited higher odds of intending to use DMHIs compared to other vendors.

All analyses were completed using Stata Statistical Software Package (47) and the G*Power application (48) to calculate preliminary power analyses.

## RESULTS

### Descriptive Characteristics of the Analytic Sample

The mean age was 23.9 years (SD 3.3), and 48.4% identified as female. Overall, 57.6% of the participants indicated an intention to use DMHIs. Regarding symptom severity, the mean PHQ-9 score was 11.3 (SD 7.5), and the mean GAD-7 score was 9.3 (SD 6.3). Just over half of the sample (51.2%) reported a perceived need for support in the past 12 months. The Chinese and Indian groups were the largest of the single-ethnicity groups, each contributing 197(12.5%) participants. Vietnamese groups and Other AANHPI participants were the smallest of single-ethnicity groups comprising 138(8.8%) and 136(8.6%) participants, respectively. Those categorized as Multi-Ethnic comprised 54 (3.4%) participants, and those categorized as Multi-Racial comprised 208 (13.2%) participants. See Table 1 for full sample descriptives.

### Multivariable Logistic Regression

Multivariable logistic regression was used to test the associations between individual-level SMHC factors and the intention to use DMHIs, controlling for symptom severity, sociodemographics, current therapy use, and vendor source. The model was statistically significant (χ^2^ (28) = 463.52, p < .001). The model explained 22% of the variance in intention to use DMHI. See Table 2 for full results.

#### Key Individual-Level SMHC Factors.

The analysis partly confirmed our main hypothesis, revealing significant associations between the intention to use DMHIs and key individual variables identified by the SMHC model. Participants who reported a *perceived need for support* over the past 12 months showed nearly twice the odds of intending to use DMHIs (OR = 1.91, CI [1.46–2.50], p < .001). Similarly, higher *mental health literacy* scores were significantly associated with increased odds of intention to use DMHI (OR = 1.14, CI [1.06–1.21], p < .001). The relationship between stigma and intention to use DMHI was more complex, however. A significant inverse relationship was found (OR = 0.63, CI [0.46–0.84], p = 0.002), indicating that individuals who reported higher perceived public stigma had higher odds of intending to use DMHIs. *Perceived self-stigma* (higher score = lower stigma) was not significantly associated with intention to use DMHI (OR = 1.31, CI [0.98–1.74], p = 0.064).

#### Sociodemographic and Other Control Variables.

Both *anxiety* and *depression* scores were included simultaneously as continuous control variables to account for symptom severity. Neither score demonstrated a significant association with the intention to use DMHIs (Anxiety: OR = 0.99, CI [0.96 = 1.03], p = 0.745; Depression: OR = 1.01, CI [0.98–1.04], p = 0.432). Individuals who were *currently in therapy* were more than twice as likely to indicate intention to use DMHIs (OR = 2.19, CI [1.59–3.00], p < .001). Those who were *more acculturated* and who were *insured* also had significantly higher odds of intending to use DMHIs (Acculturation: OR = 1.43, CI [1.12–1.84], p = 0.004; Insured: OR = 1.71, CI [1.13–2.61], p = 0.012).

#### Ethnicity.

Using the Chinese group as the reference, several ethnic groups showed significantly higher odds of intending to use DMHIs. *Indian* and *Filipino* participants were both nearly twice as likely as Chinese participants to indicate intention to use DMHIs (Indian: OR = 1.78, CI [1.15–2.73], p = 0.009; Filipino: OR = 1.75, CI [1.11–2.76], p = 0.016). People in the “*Other AANHPI*” group also showed significant intention to use DMHIs (OR = 1.72, CI [1.07–2.79], p = 0.026)

Sensitivity analyses excluding Vendor 3. Sensitivity analyses largely supported the robustness of the main analysis with a few changes of note. In our main analysis, *Japanese* participants showed the highest odds of indicating intention to use DMHIs, and were more than three times as likely as *Chinese* participants to indicate intention (OR = 3.37, CI [1.71–6.62], p < 0.001). While Japanese participants still showed higher intention than Chinese participants in our sensitivity analyses, the results were not statistically significant (p = .055) and odds were reduced to 2.11 (CI [0.98–4.53]). This is worth noting, as Vendor 3 accounted for a majority [113 out of 147, or 77%] of Japanese participants in the sample. Despite this variation, our core findings of interest remained consistent, demonstrating the robustness of the data despite discrepancies resulting from Vendor 3 sampling (see Table 3 in supplemental materials for a detailed comparison of full and restricted models).

## DISCUSSION

This manuscript addressed the primary aim by utilizing the SMHC model to quantitatively assess key individual-level factors associated with the intention to use DMHIs among AANHPI emerging adults,. The strong association of key SMHC variables suggests that established help-seeking theories can be applied effectively in the digital context.

### Primary Drivers of Digital Help-Seeking Intent

The findings validate our main hypothesis, showing that perceived need for support (OR = 1.91) and mental health literacy (OR = 1.14) are significantly associated with intention to use DMHI. This is significant because AANHPI can often exhibit a lack of perceived need for mental health support due to cultural norms like stoicism or the desire to preserve an appearance of wellness (49). For AANHPI, accurately recognizing the need for support and when to seek support for mental health is a critical first step, particularly with digital resources. In our other study (manuscript in preparation), we found that in addition to symptoms of depression and anxiety, mental health literacy was significantly associated with having a perceived need for mental health support. Furthermore, increasing mental health literacy may be a vital pathway to promoting DMHI adoption, as individuals with better knowledge of symptoms and resources are more inclined to intend to seek support (50, 51).

Values and experiences that are shared across ethnic groups can shape how AANHPI understand mental health and choose to seek help. Certain shared norms across AANHPI groups–such as collectivism, respect for authority and elders, and the prioritization of professional or academic achievement–while at times considered protective factors, can also result in minimizing symptoms of mental illness in order to preserve an appearance of wellness or to avoid perceived consequences (52, 53). Similarly, expectations of self-control or stoicism can often result in the downplaying of emotions and a lack of perceived need for mental health support (54). A common desire across many AANHPI people is to avoid burdening others (55), which can lead to less help-seeking behavior and greater attitudes of taking on problems alone, limiting the early identification of emerging mental illness. AANHPI emerging adults also often navigate bicultural identities, balancing the values of their AANHPI culture with the values of mainstream U.S. culture, and resulting in a sense of exclusion from both cultures (56, 54). Expectations tied to the “model minority” stereotype can also result in individuals’ underreporting distress (8) based on a belief that they should not be experiencing any mental distress. Stigma and the fear of judgment from family and community when seeking help for mental illness can negatively affect attitudes toward care (55).

### The Complex Role of Stigma

The results revealed complex associations with perceived stigma. Personal attitudes about mental health treatment were not significantly associated with the intention to use DMHIs (p = 0.064). On the other hand, individuals who reported higher levels of perceived public stigma were more likely to intend to use DMHIs. This counterintuitive finding aligns with the unique barriers faced by AANHPI individuals, where the fear of judgment from family and community negatively affects traditional help-seeking attitudes. DMHIs offer specific advantages such as anonymity and privacy, potentially making them the preferred avenue for those who perceive significant external risk (high public stigma) associated with seeking mental health support through visible means. This suggests that for AANHPI emerging adults, DMHIs function as a stigma-avoidance mechanism for mental health help-seeking.

### Influence of Therapy Engagement and Acculturation

The strongest single correlate of DMHI intention was current therapy use (OR = 2.19). This suggests that DMHIs are currently favored by those already engaged in the help-seeking process or familiar with formal treatment. It challenges the idea that DMHIs primarily serve to draw in individuals entirely new to mental health support by serving as an “on-ramp” to therapy, indicating they might instead serve as effective adjuncts or supplemental tools for those already on a treatment pathway. Similarly, the significant association with high acculturation (OR = 1.44) reinforces the idea that individuals more integrated into mainstream U.S. culture may be more receptive to both formal treatment and digital solutions.

### Symptom Severity

The absence of a significant association between anxiety or depression severity and DMHI intent, when controlling for all other factors, suggests that the subjective recognition of distress (perceived need) is a more powerful predictor of digital help-seeking intention than more objective measures such as the PHQ-9 and GAD-7. These results are in line with the SMHC model’s conceptualization of symptom severity, which was seen as a precursor or antecedent to a more subjective sense of illness, or perceived need.

### Ethnic heterogeneity

A strength of this paper is that it included an ethnically diverse set of AANHPI emerging adults. Significant ethnic variations that were observed—with Indian, Filipino, and "Other" AANHPI groups exhibiting higher odds of intention compared to the Chinese group—show the importance of moving beyond an aggregate AANHPI category. While this study was largely exploratory in nature, these differences suggest that even drivers of digital help-seeking vary considerably across ethnic subgroups, likely reflecting unique sociocultural experiences, patterns of technology use, levels of acculturation, or specific migration-related stressors. Ethnic aggregation risks ‘flattening’ these key differences and may lead to missed opportunities to reach members of ethnic groups that may already be open to utilizing digital support. Future research should also consider how to better reach smaller and less represented AANHPI ethnic groups, such as Laotian, Hmong, or Samoan people. The significantly higher odds of DMHI intention shown by those aggregated in the “Other” AANHPI category is perhaps a signal of an opportunity to serve the mental health of lesser represented cultures.

AANHPI ethnic groups also have unique sociocultural experiences that can shape their approach to mental health. For example, certain groups such as Vietnamese or Native Hawaiian and Pacific Islander (NHPI) communities face migration- or displacement-related stressors that increase their chances of developing mental health issues, while also limiting their access to familiar and appropriate channels of support (16). Ethnic groups that have experienced colonization–such as Taiwanese, Indians, or Filipinos–may harbor a mistrust in health systems due to repeated experiences of health inequities (57, 58), and a loss of identity that may limit their likelihood to seek support (59). Displacement and colonization can lead to intergenerational trauma, increasing mental health issues and reducing formal help-seeking even among later generations that have not directly experienced such stressors (58, 60). These unique ethnocultural experiences influence the mental health experience of AANHPI emerging adults.

### Limitations

Although this study did validate the use of the SMHC model to identify key factors associated with the intention to use digital tools for mental health, these findings should be interpreted in light of several limitations.

#### Measurement and scope.

This study relied primarily on self-reported survey data which, while convenient, may result in certain biases such as recall bias or social desirability. This can affect key independent variables such as measures around stigma, mental health literacy, perceived need over the past 12 months, or the intention to use DMHIs. In addition, the construct validity of the instruments warrants consideration. For example, we used a simplified dichotomous proxy for acculturation based on English language use and preference. While common in large scale surveys, this measurement fails to capture the complex nature of acculturation (61, 62). Likewise, DMHIs were broadly defined as any digital tool used for mental health support, limiting our ability to explore more nuanced questions such as if certain technologies may be more widely acceptable than others. Furthermore, our definition of digital tools did not explicitly inquire about interest in using artificial intelligence (AI), which is an increasingly popular tool for mental health support; a limitation that epitomizes the fast-paced nature of the field of digital technology.

#### Mental health conditions.

Our investigation focused on an analysis of anxiety and depression symptoms. Although these symptoms as measured did not seem to be associated with intent, these findings may be different for those with other severe or chronic mental illness. Future studies should replicate the application of the SMHC model to a broader range of mental health challenges.

#### Sampling and generalizability.

The data for this investigation were from a cross-sectional convenience sample that was collected via online survey panels (Qualtrics). While this methodology allowed for the collection of real-world DMHI data, it also likely introduced self-selection bias, likely biasing towards individuals who are more tech-savvy, acculturated, motivated to participate in mental health research. Thus, the generalizability of our findings is limited. In addition, the reliance of multiple vendors introduced significant, uncontrolled variation in several variables, although we did control for vendor source in our main analysis and conducted a sensitivity analysis that suggested that our findings were largely robust even with the exclusion of Vendor 3. Future efforts should utilize strategies such as probability-based sampling that are representative of the general population, or methods that involve collecting data from vetted sampling sources that are able to reach a variety of AANHPI subgroups, including those from hard-to-engage ethnic groups.

### Implications and Future Directions

Despite the aforementioned limitations, there are several opportunities that establish a foundation for future research. Findings from our analysis suggest the potential for the SMHC model to identify key factors associated with the intention to use digital mental health among AANHPI emerging adults. Future research should build upon this foundation, moving beyond this initial scope to address limitations, explore other help-seeking theories, advance theoretical integration, and maintain pace with fast-evolving technologies.

#### Improving sampling and measurement.

As mentioned, targeted recruitment strategies such as nationally representative probability-based sampling and leveraging vetted sample sources can be used to improve the generalizability of future work. Future work may also benefit from using more detailed measures of key outcomes and predictors such as more nuanced measures of DMHI intent, or validated instruments that measure perceived need for mental health support. Validating existing measures for use among AANHPI emerging adults can also help with strengthening future work with this population.

#### Moving from DMHI intention to DMHI utilization.

Guided by the SMHC model, this work identified key factors associated with intention to use DMHI, an important antecedent to the behavior of DMHI use. Future work should expand upon this and explore the application of the SMHC model to the use of and adoption of DMHI, which remains low in real-world settings. Longitudinal studies should track participants over time to identify what factors successfully bridge the gap between intention to use digital tools and the actual use of digital tools.

While the SMHC model provides an excellent starting point to framing digital help-seeking through personal and attitudinal factors, mental health help-seeking is inherently episodic, systemic, and social. To more fully understand DMHI adoption, especially among AANHPI emerging adults for whom social norms and social circles often influence preferences for mental health support (16), future studies should integrate external socioecological factors. Specifically, future research should incorporate additional, established frameworks of help-seeking, such as the Network Episode Model (63), which frames how social circles can influence an individual’s pathways to care. Frameworks that incorporate the Diffusion of Innovations (64) may also be useful in understanding how dissemination of mental health technologies may or may not be happening. Likewise, more recent frameworks such as the Comprehensive Model for Mental Health Access, or CoMMA (65), provide a modern framework that incorporates ideas for how technology integrates at different points in the help-seeking process. Additionally, future work should leverage qualitative or mixed methods to capture nuances of how individual and sociocultural factors intersect to influence mental health care decision-making among AANHPI. Moving towards an updated model that accounts for the digital and social influence of today’s world, allowing for improved implementation and understanding new avenues of intervention to better support mental health.

Finally, there is a need to deepen our understanding of DMHI’s role, or perhaps *roles*, in the mental health continuum. In our current sample, DMHIs are more likely to be used by those already engaged in therapy. This suggests that for some, DMHIs serve as adjuncts or supplemental tools rather than primarily serving individuals entirely new to support. Future studies should differentiate between different types of digital tools (e.g., wellness apps vs. AI chatbots) and investigate whether certain types are more useful at different stages of the help-seeking continuum. For example, certain tools may better aid those with lower mental health literacy to identify symptom identification. Studying the role of DMHI can help create a framework that leads to a more targeted recommendation of digital tools, based on user need and their place in the help-seeking continuum. This is particularly salient given the emergence of cutting edge technology such as AI-based chatbots, whose public adoption and perception require further exploration within the AANHPI community.

## CONCLUSION

The findings from this manuscript support the use of a help-seeking model, specifically the SMHC model, in identifying key individual predictors of digital help-seeking intention among AANHPI emerging adults. This work is critical for informing interventions aimed at addressing mental health disparities by clarifying that efforts to increase DMHI adoption should focus on promoting mental health literacy, thus improving the accurate identification of a perceived need for support. These quantitative findings establish the necessary foundation for the subsequent papers investigating network-level factors and providing qualitative context. The overall project aims to provide a comprehensive understanding of the mechanisms driving or inhibiting digital engagement across this diverse population.

## Supplementary Material

This is a list of supplementary files associated with this preprint. Click to download.

• SUPPLEMENTALMATERIALS.docx

## Figures and Tables

**Table 1:** Sample characteristics

SAMPLE CHARACTERISTIC (N = 1,577)	OVERALL % or $\bar{X}$ (SD)
**Intention to Use DMHI, Yes (Outcome)**	908 (57.6%)
**Sociodemographics**	
Age	23.9 (3.3)
Sex, Female	763 (48.4%)
Education level, College degree or higher	967 (61.3%)
Employment status, Employed	926 (58.7%)
Health insurance, Insured	1454 (92.2%)
Acculturation, English spoken at home	935 (59.3%)
Ethnicity	
*Chinese*	197 (12.5%)
*Filipino*	165 (10.5%)
*Indian*	197 (12.5%)
*Japanese*	147 (9.3%)
*Korean*	150 (9.5%)
*NHPI*	185 (11.7%)
*Vietnamese*	138 (8.8%)
*Other*	136 (8.6%)
*Multi-Ethnic*	54 (3.4%)
*Multi-Racial*	208 (13.2%)
**Individual-Level Variables (SMHC Model)**	
Perceived need for support, Yes	807 (51.2%)
Mental health literacy	5.2 (1.9)
Perceived self-stigma	36.1 (5.4)
Perceived public-stigma	29.9 (4.7)
**Mental Health Experience**	
PHQ-9	11.3 (7.5)
GAD-7	9.3 (6.3)
Current Therapy Use, Yes	464 (29.4%)
**Other Variables**	
Sub-vendor	
*Vendor 1*	713 (45.2%)
*Vendor 2*	490 (31.1%)
*Vendor 3*	374 (23.7%)

**Table 2:** Results of multivariable logistic regression (N = 1,577)

DV: Intention to use DMHI (Y/N)	OR	SE	z	p-value	95% conf. interval	
					*LL*	*UL*
Individual-Level SMHC Variables						
Perceived Need	1.91	0.26	4.68	0.000	1.46	2.50
Mental health literacy score	1.14	0.04	3.83	0.000	1.06	1.21
Self-stigma	1.31	0.19	1.85	0.064	0.98	1.74
Public stigma	0.63	0.10	−3.07	0.002	0.46	0.84
Control Variables						
Anxiety score	0.99	0.02	−0.33	0.745	0.96	1.03
Depression score	1.01	0.01	0.79	0.432	0.98	1.04
Sex (F)	1.13	0.15	0.92	0.358	0.87	1.45
Age	1.05	0.02	2.44	0.015	1.01	1.09
College degree or higher	1.35	0.19	2.15	0.032	1.03	1.78
Currently working	0.90	0.12	−0.85	0.397	0.70	1.15
Insured	1.71	0.37	2.52	0.012	1.13	2.61
High acculturation	1.43	0.18	2.85	0.004	1.12	1.84
Currently in therapy	2.19	0.35	4.85	0.000	1.59	3.00
Vendor (Vendor 1 comparison group)						
*Vendor 2*	0.88	0.11	−0.99	0.323	0.68	1.13
*Vendor 3*	3.97	1.08	5.07	0.000	2.33	6.76
Ethnicity (Chinese comparison group)						
*Filipino*	1.75	0.41	2.42	0.016	1.11	2.76
*Indian*	1.78	0.39	2.61	0.009	1.15	2.73
*Japanese*	3.37	1.16	3.52	0.000	1.71	6.62
*Korean*	0.98	0.26	−0.08	0.933	0.58	1.65
*NHPI*	1.57	0.50	1.42	0.156	0.84	2.94
*Vietnamese*	1.15	0.28	0.57	0.566	0.72	1.84
*Other*	1.72	0.42	2.22	0.026	1.07	2.79
*Multi-ethnic*	0.77	0.26	−0.75	0.453	0.40	1.51
*Multi-racial AANHPI*	1.12	0.25	0.48	0.632	0.71	1.74
Constant	0.04	0.03	−4.25	0.000	0.01	0.19
Model Fit Statistics						
Model χ^2^ = 463.52, p < .001						
Pseudo R^2^ = 0.22						

## Abbreviations

AANHPI: Asian American, Native Hawaiian, Pacific Islander
DMHI: Digital Mental Health Intervention
SMHC: Seeking Mental Health Care Model
PHQ: Patient Health Questionnaire
GAD: Generalized Anxiety Disorder Questionnaire
MHL: Mental Health Literacy
